# Cellular Automata Modeling of Three-Dimensional Chitosan-Based Aerogels Fiberous Structures with Bezier Curves

**DOI:** 10.3390/polym13152511

**Published:** 2021-07-30

**Authors:** Igor Lebedev, Daria Lovskaya, Maria Mochalova, Igor Mitrofanov, Natalia Menshutina

**Affiliations:** Science and Education Center for Transfer of Biopharmaceutical Technologies, Mendeleev University of Chemical Technology of Russia, 125047 Moscow, Russia; igor170491@yandex.ru (I.L.); daria.lovskaya@gmail.com (D.L.); mochalovamarie@yandex.ru (M.M.); ivmitrofanov@gmail.com (I.M.)

**Keywords:** aerogels, chitosan, cellular automata, modeling, parallel computing, fibrous structure

## Abstract

In this work, a cellular automata approach was investigated for modeling three-dimensional fibrous nanoporous aerogel structures. A model for the generation of fibrous structures using the Bezier curves is proposed. Experimental chitosan-based aerogel particles were obtained for which analytical studies of the structural characteristics were carried out. The data obtained were used to generate digital copies of chitosan-based aerogel structures and to assess the accuracy of the developed model. The obtained digital copies of chitosan-based aerogel structures will be used to create digital copies of aerogel structures with embedded active pharmaceutical ingredients (APIs) and further predict the release of APIs from these structures.

## 1. Introduction

One of the most important problems in the pharmaceutical industry is the urgent need for new functional materials for the creation of drug delivery systems, including targeted delivery to the brain. The transition to new methods of drug delivery using new materials and technologies opens up great prospects for the pharmaceutical industry. Aerogels are a prime example of such materials, since they have such unique properties as high specific surface area (200–2000 m^2^/g), high porosity (up to 99%), and small pore size (2–50 nm) [1]. The possibility of obtaining aerogels based on substances approved for use in the pharmaceutical industry opens up the possibility of creating innovative materials with specified characteristics that can be used as drug carriers, as well as fundamentally new drug delivery systems [2,3].

Aerogels have a mesoporous structure—they contain an open pores network with a diameter of 2 to 50 nm. The aerogel framework in most cases is a cluster formed by spherical nanosized primary globules. The size of the globules depends on the aerogel material and conditions of experiment. The primary globules form the secondary ones, secondary globules are aggregated into tertiary globules. Thus, the aerogel framework is a self-similar structure. However, the structure of aerogels may not consist of globules. At the moment, aerogels with a structure consisting of nanofibers, created on the basis of biopolymers such as cellulose and chitosan, are of increasing interest [4,5,6].

Chitosan-based aerogels are of growing interest in this field. The biopolymer chitosan is a natural polysaccharide that is safe for humans. Chitosan is obtained by deacylation chitin, which is located in the cell walls of fungal cells, shells of crustaceans, and insects. Chitosan is the second most abundant polysaccharide derived from biomass, while it has a fairly low cost and is an environmentally friendly product. Chitosan is used as matrix for smart bionanocomposites. In [7], cationic chitosan was used to obtain nanocarriers with controlled release properties. In [8], bionanocomposites consisting of potato starch, chitosan, and graphene oxide showed different properties useful for various applications, particularly in food products packaging materials.

Chitosan is biocompatible, nontoxic, and can bind some organic compounds. Chitosan nanoparticles reduce enzymatic drugs degradation and non-targeted tissues damage. The aforementioned features of chitosan allow it to be widely used in pharmaceuticals, including for the development of new drug delivery systems [9].

The structure and properties of aerogels depend on the process of their obtaining. Aerogels based on the same material can have different characteristics. Thus, the problem of obtaining an aerogel with specific properties is always associated with carrying out a large number of experiments, which significantly increases the research time and cost. This problem can be solved by using methods of computer and mathematical modeling, which will allow for obtaining digital copies of aerogel structures and predict their properties—creating “digital twins”. “Digital twins” will make it possible to partially replace a full-scale experiments with a computational ones, which will significantly reduce the time and cost of research in the development of new aerogels with the required properties.

Today, the cellular automata (CA) approach is widely used for modeling nanostructured porous materials such as aerogels.

Cellular automata is one of the modern approaches to modeling, which is based on simplification and discretization of the system. The basic principle of the CA-approach is dividing the system into identical cells, which, at each moment of time have one of a given states, while the rules for the transition of a cell from one state to another are determined. So, the studied system is represented as a set of cells interacting with each other. The property of the cellular automata is the locality of the transition rules from. Thus, the system can be described using simple calculations, and also does not require a mathematical description of the entire system behavior. It allows simulation processes and phenomena that are described by ordinary differential equations (ODE) or those systems that have not yet been described using ODE. Thus, cellular automata describes the behavior of large and complex systems using simple transition rules. In addition, CA-based models can be easily implemented using high-performance parallel calculations, which further increases the computational efficiency [10,11,12,13,14,15,16,17].

Currently, CA-models are widely used in various fields of science, including chemistry and chemical technology, in porous nanomaterials modeling. Among porous materials structures models, aggregation models such as diffusion-limited aggregation (DLA) [18] and diffusion-limited cluster aggregation (DLCA) are widely used [19,20].

**Figure 1:**
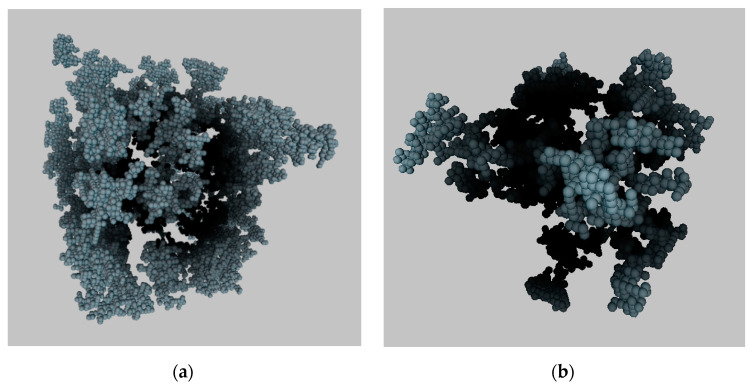
Digital 3D aerogels structure, generated with aggregation models: (**a**) DLA; (**b**) DLCA.

DLA and DLCA models belong to two classes of aggregation models—particle-cluster aggregation and cluster-cluster aggregation, respectively. They can be modified, allowing changing the method of generation to more accurate structure generation for a specific material. Figure 1 shows digital three-dimensional (3D) porous structures obtained using the DLA and DLCA models. The size of these structures is 80 × 80 × 80 cells and the scale of one cell is 2 nm.

These models allow generating of aerogels digital copies, formed by irreversible aggregation [21,22]. However, aggregation models are not suitable for modeling fibrous aerogels such as chitosan-based aerogels. It seems promising to use Bezier curves for modeling the structure of fibrous materials.

**Figure 2:**
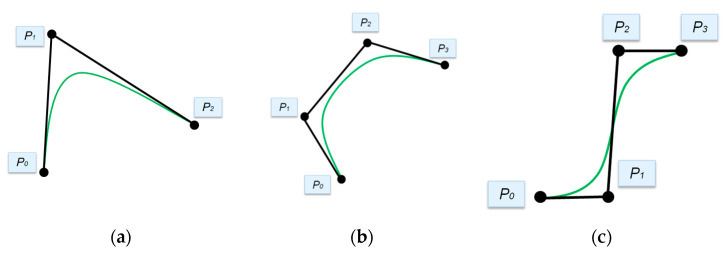
Bezier curve examples: (**a**) Second-order curve; (**b**) Third-order curve; (**c**) Third-order curve with another control point configuration.

Bezier curves were first introduced in 1962. They are traced using control points. The number of control points can be two, three, four or more and determines the order of the curve and sets its curvature: two control points define a Bezier curve as a linear curve (first-order curve, straight line), three control points as quadratic (second-order curve), four as cubic (third-order curve), etc. Figure 2 shows the Bezier curves of the second and third order with different locations of the control points.

Initially, Bezier curves were used in computer-aided design of car bodies. Currently, due to their simplicity and the ability to accurately control the shape of the curve by varying the coordinates of the control points, they are widely used in approximation problems [23,24]. Bezier curves are used for calculating motion trajectories [25]. In [26], Bezier curves were used to optimize the path finding of artificial intelligence.

In addition, Bezier curves are used in the design of structures that contain developable surfaces because Bezier curves give a formal description of complex shapes for subsequent computer processing [27].

In the proposed model, Bezier curves are used to create digital copies of the fibers, which shape corresponds to the real fiber.

## 2. Materials and Methods

### 2.1. Synthesis of Chitosan-Based Gel Particles via Dripping Method

In this work, the process of obtaining aerogel particles based on chitosan was investigated. This process includes the following main stages: preparation of an initial solution of chitosan in the acetic acid, preparation of a cross-linking agent, formation of a chitosan gel, multistep solvent exchange of an aqueous solvent with alcohol, and supercritical drying. To prepare the initial solutions, chitosan with molecular weight 111 kD (Sigma-Aldrich, Saint Louis, MO, USA) and chemically pure acetic acid (Sigma-Aldrich) were used. Chemically pure sodium hydroxide (Sigma-Aldrich) was used as a crosslinking agent for the formation of the gel particles. To obtain chitosan-based gel particles, the dripping method was used as described in [28]. In the course of the study, such parameters as the concentration of the initial solution of chitosan (1 wt% and 2 wt%), the concentration of acetic acid (0.1 M and 0.2 M), and the concentration of sodium hydroxide (0.1 M and 1 M) were varied. In addition, samples were additionally obtained with the addition of a third-party polymer for functionalization of the finished material (in this work, we used a pluronic polymer, 0.05 wt%).

**Figure 3:**
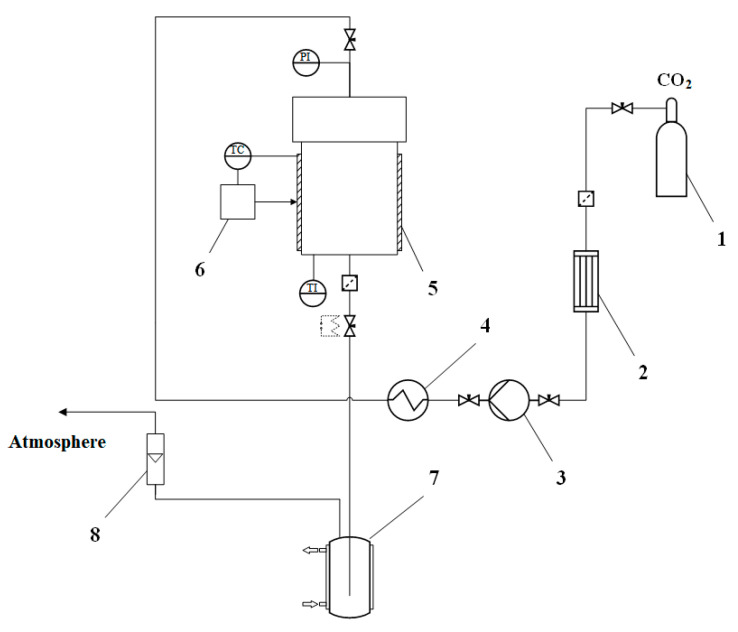
Diagram of a supercritical drying plant: 1—carbon dioxide vessel; 2—condenser, 3—high pressure piston pump, 4—thermostat, 5—high pressure apparatus; 6—thermal control system, 7—separator with a cooling jacket, 8—flow meter, PI—pressure gauge, TC—temperature sensor, TI—temperature sensor.

The supercritical drying process was carried out as described in [29,30]. The supercritical drying plant diagram is presented in the Figure 3.

The supply of carbon dioxide is carried out from the vessel 1 to the condenser 2. In the condenser 2, in order to avoid the phase transition of carbon dioxide, it is cooled to 5 °C. Then the pressure is generated by high pressure piston pump 3. Further, carbon dioxide enters thermostat 4, after that supercritical carbon dioxide enters the high-pressure apparatus 5. The high-pressure apparatus contains: a thermostat, a pressure gauge, and a temperature sensor. The process of heating the apparatus takes place with the help of a flexible heating tape placed over the body. The regulation of the carbon dioxide consumption is carried out by a system of valves at the outlet of the apparatus. The flow rate of carbon dioxide gas is displayed on a flow meter. Chitosan-based gel particles in envelopes are placed in a high-pressure apparatus, 10 g of isopropyl alcohol is preliminarily poured into it. Then, the apparatus is sealed. The supply of liquid carbon dioxide is carried out into the apparatus, and with the pump, a pressure of 120 atm is generated, the apparatus is heated up to a temperature of 40 °C. Liquid carbon dioxide goes into a supercritical state and a homogeneous mixture with isopropyl alcohol is formed. Then the solvent is displaced from the free volume of the apparatus for 1 h at a pressure of 120 atm, a temperature of 40 °C, a carbon dioxide consumption of 300 nl/h. The next stage is the diffusion replacement of the solvent in the pores of the gel with supercritical carbon dioxide, which takes place for 5 h at a pressure of 120 atm, a temperature of 40 °C, and a flow rate of carbon dioxide of 100 nl/h. After drying, the supply of carbon dioxide is shut off and the pressure is released from the high-pressure apparatus at a rate of 4 atm/min. Upon reaching the atmospheric pressure, the apparatus is depressurized, the obtained aerogel samples are removed from the apparatus, and the drying process is completed.

Process parameters were: temperature 40 °C, pressure 12–14 MPa. Drying time was 6 h.

### 2.2. Analytical Experiments

The textural characterization of the obtained aerogels was carried out by low-temperature N_2_ adsorption–desorption analysis (ASAP 2020MP, Micromeritics, Norcross, GA, USA). Samples were preliminary dried under a vacuum at 50 °C for 20 h. Specific surface area was determined by the BET method. BJH analysis was used to determine the average pore diameter of aerogel particles using desorption techniques. Aerogel shape and appearance were analyzed using SEM (JEOL 1610LV, JEOL Ltd., Tokyo, Japan). Samples were platinum-sputtered prior to imaging in order to minimize charging and improve the image contrast.

To measure the bulk density of a chitosan-based aerogel particle, the following method was used: the measurement is carried out using a caliper, a Petri dish, and analytical balance. Twenty particles are selected and the diameter of each particle is measured. Based on the particle diameter, its volume is calculated using the following:

(1) $$V_{\mathrm{particle}}=\frac{4\pi {\left(d/2\right)}^{3}}{3},$$

where $V_{\mathrm{particle}}$—particle volume, m^3^, d—particle diameter, m.

Then the Petri dish is weighed on an analytical balance, its weight is zeroed. 20 particles placed into it and weigh—total mass m_20_ of 20 particles is obtained. Then the particle bulk density is calculated by the follows:

(2) $$\rho_{\mathrm{bulk}}=\frac{m_{20}}{\sum_{i=1}^{20}V_{\mathrm{particle},i}},$$

where $\rho_{\mathrm{bulk}}$—particle bulk density, kg/m^3^, m_20_—total mass of 20 particles, kg, $V_{\mathrm{particle},i}$—volume of a single particle, m^3^.

Skeletal density $\rho_{\mathrm{skeletal}}$ was obtained using He pycnometry.

The porosity of the particles was calculated with its skeletal and bulk densities:

(3) $$\mathrm{Porosity}=1-\frac{\rho_{\mathrm{bulk}}}{\rho_{\mathrm{skeletal}}\;}\cdot 100,$$

where Porosity—porosity, %, $\rho_{\mathrm{skeletal}}$—particle skeletal density, kg/m^3^.

16 samples of chitosan-based aerogel particles were obtained and analyzed. Table 1 shows the process parameters that were varied.

**Figure 4:**
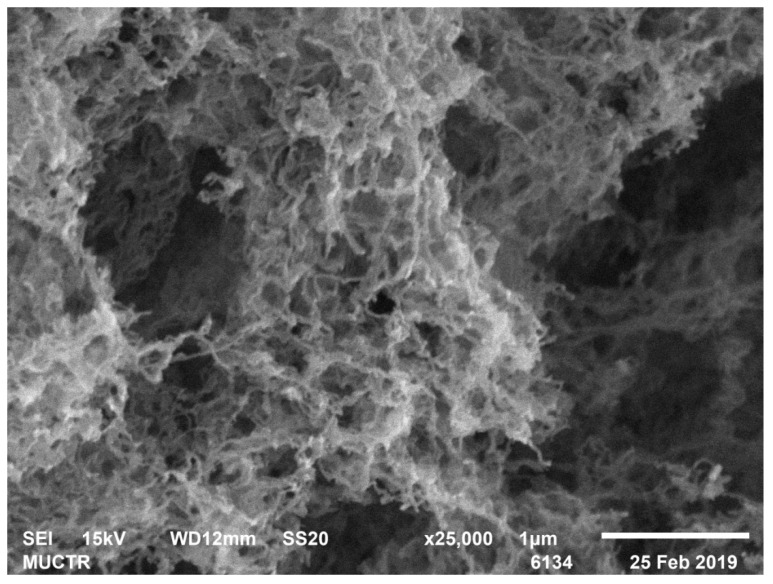
SEM-image of sample 2 chitosan-based aerogel particles.

**Figure 5:**
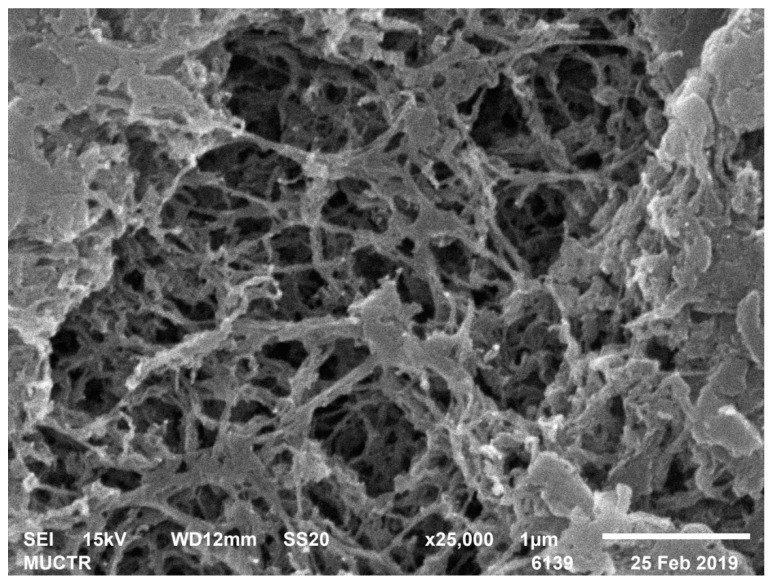
SEM-image of sample 3 chitosan-based aerogel particles.

**Figure 6:**
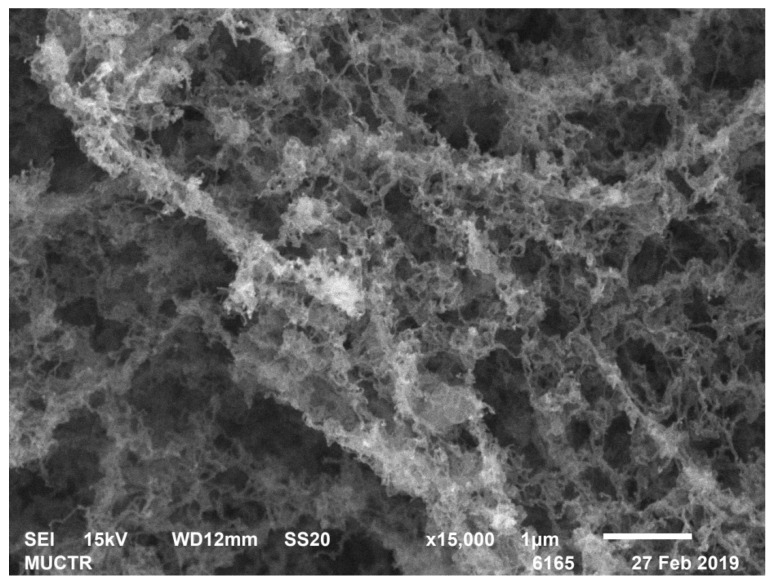
SEM-image of sample 6 chitosan-based aerogel particles.

**Figure 7:**
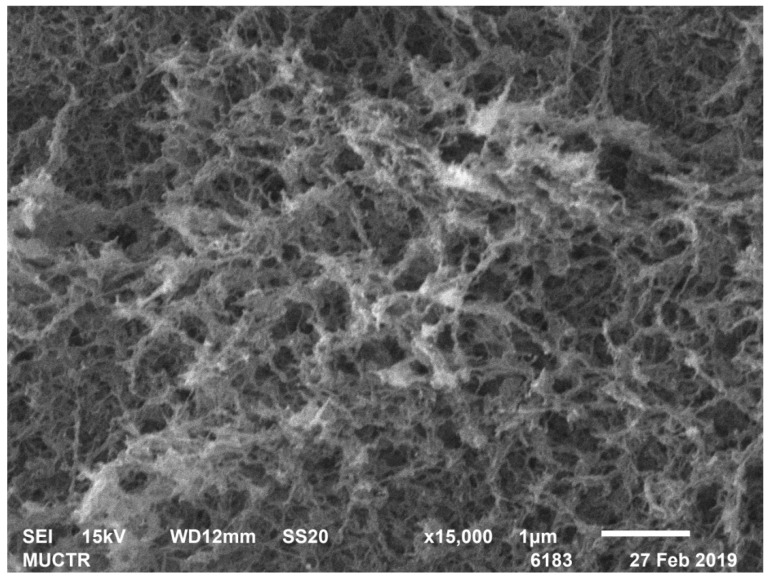
SEM-image of sample 8 chitosan-based aerogel particles.

**Figure 8:**
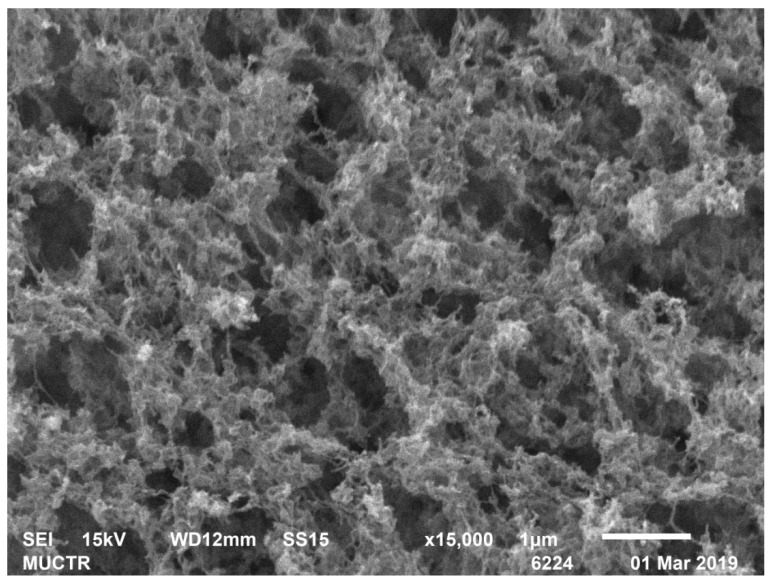
SEM-image of sample 11 chitosan-based aerogel particles.

**Figure 9:**
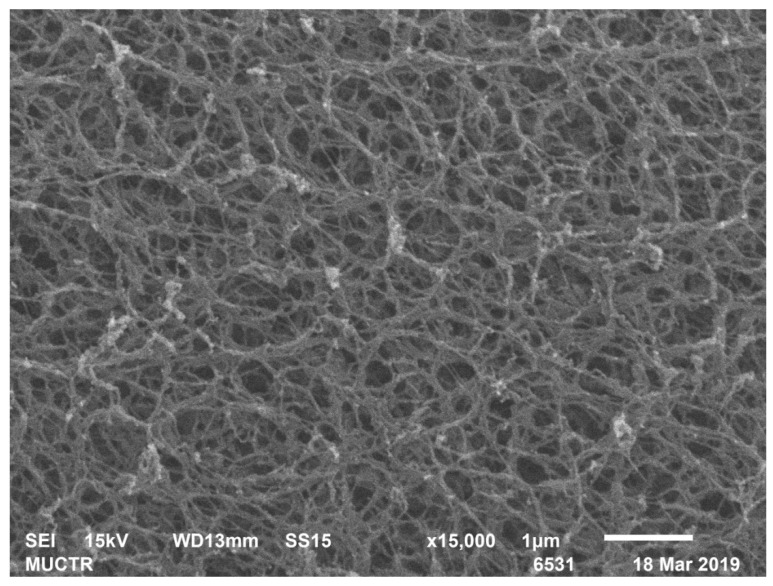
SEM-image of sample 15 chitosan-based aerogel particles.

Table 2 shows the results of analytical studies of the samples: Specific surface area S_BET_, m^2^/g, total mesopore volume V_pore_, cm^3^/g, average pore diameter D_pore_, nm, bulk ρ_bulk_ and true ρ_true_ particle density, kg/m^3^. Figure 4, Figure 5, Figure 6, Figure 7, Figure 8 and Figure 9 show SEM-images of chitosan-based aerogel particles samples.

As can be seen from Figure 4, Figure 5, Figure 6, Figure 7, Figure 8 and Figure 9, chitosan-based aerogels have a fibrous structure, which cannot be represented by a set of spherical globules. In this work, a new approach for obtaining digital copies of the structures of fibrous aerogels, using a cellular automata approach that uses Bezier curves for generation, is proposed.

The obtained samples and the results of analytical studies were used to generate digital copies of porous fibrous nanostructures.

## 3. Theory

### 3.1. Fibrous Nanostructure Model

To generate digital copies of chitosan-based aerogels samples, which were obtained earlier in Section 2, a model based on Bezier curves was developed. The main idea of the proposed model for digital copies of fibrous nanostructures generating is following: Bezier curves are randomly plotted in the field, then aerogel fibers are generated in those places where the curves pass. Thus, the model makes it possible to obtain a structure consisting of intersecting fibers, corresponding to the real ones.

The model construct third-order Bezier curves according to the following expression:

(4) $$B\left(t\right)={\left(1-t\right)}^{3}P_{0}+3t{\left(1-t\right)}^{2}P_{1}+3t^{2}\left(1-t\right)P_{2}+t^{3}P_{3},\;t\in \left[0,1\right],$$

where $P_{0},P_{1},P_{2},P_{3}$—control points, which contain the set on three coordinates.

**Figure 10:**
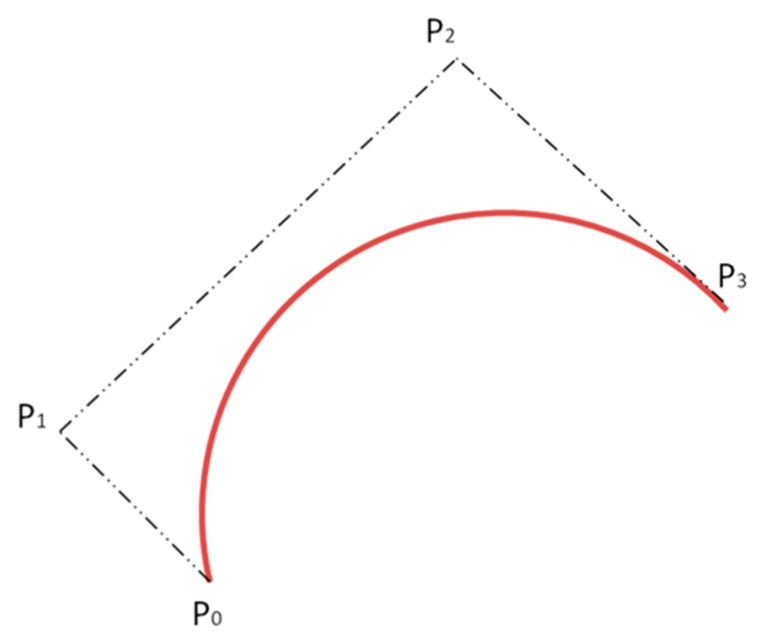
Third-order Bezier curve.

According to Equation (4), the curve is plotted using four control points. $P_{0}$ and $P_{3}$ are the beginning and end of the curve. $P_{0}$ and $P_{3}$ are randomly selected on different edges of the field. $P_{1}$ and $P_{2}$ are random points on the field between $P_{0}$ and $P_{3}$. In this case, the curve passes through points $P_{0}$ and $P_{3}$, but it does not pass through points $P_{1}$ and $P_{2}$ (Figure 10).

The third-order Bezier curve was chosen since the second-order curve does not have sufficient curvature to reflect the shape of a real fiber, and it curves with an order greater than the third require a very large amount of computation.

The model uses the porosity of the aerogel and the cross-sectional diameter (diameter) of the fiber as input parameters. The diameter of the digital fiber matches the fiber diameter of the real structure.

**Figure 11:**
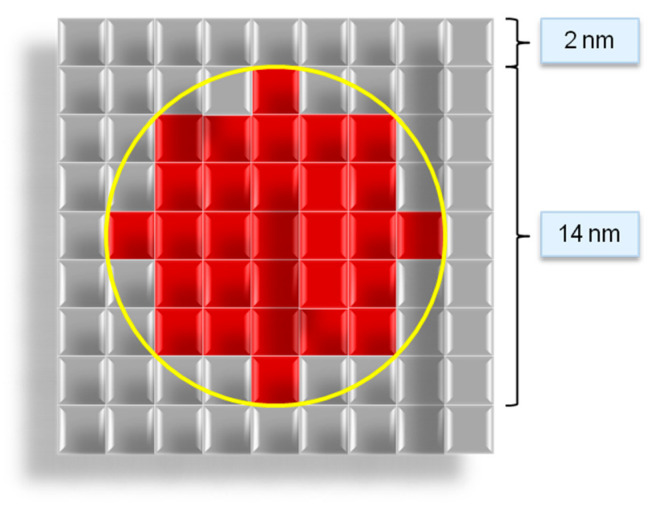
Cross-section of a digital representation of a 14 nm fiber with a 2 nm cell scale.

Developed model has the following assumption:

- The modeling space is divided into cells of the same size, square (two-dimensional structure) or cubic (three-dimensional structure) shapes. The scale of one cell is an integer and is specified in nanometers.
- Each cell can be in one of two given states: “fiber” and “empty”.
- Neighboring cells are cells that have a common face.
- Cells have a “fiber” state if they lie in the path of the Bezier curve.
- All fibers have the same diameter.
- The fiber diameter of the digital structure is set by a certain number of cells. Figure 11 shows an example of a fiber cross-section as a set of cells at a scale of one cell of 2 nm.
- The beginning and end of the fiber lie on the edges of the modeling space.

Initially, the control points $P_{0}$, $P_{1}$, $P_{2}$ and $P_{3}$ are randomly selected on the field, $P_{0}$ and $P_{3}$ lie on one of the edges of the field. A Bezier curve is constructed from these control points in accordance with (1). Bezier curve passes through the points $P_{0}$ and $P_{3}$ and does not pass through points $P_{1}$ and $P_{2}$. Cells which lie on the curve pass are set into "fiber" state. Further, the fiber diameter increases until it reaches the specified one. New fibers are generated until the structure reaches the specified porosity.

**Figure 12:**
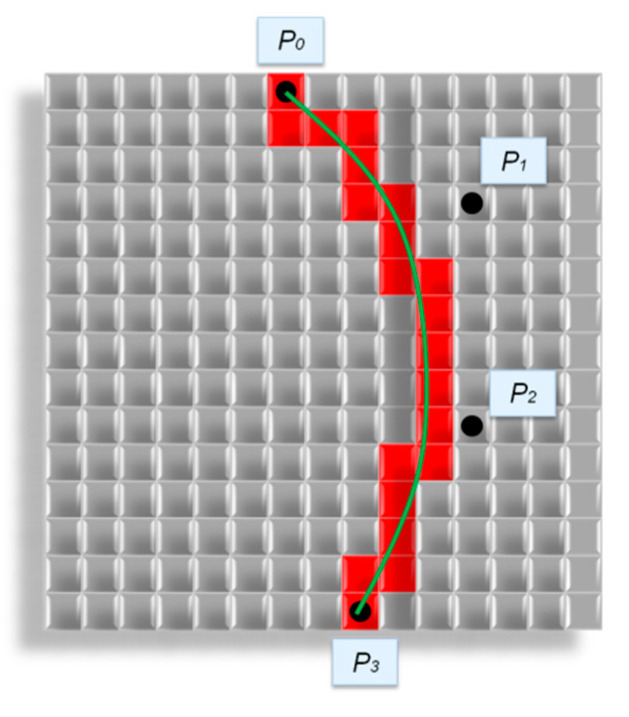
Creation a fiber with Bezier curve: 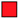
—“fiber” cells, 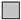
—“empty” cells, ⚫—control points, 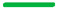
—Bezier curves.

Figure 12 shows the constructing a fiber along the Bezier curve.

Figure 12 shows the control points $P_{0},P_{1},P_{2},P_{3}$ selected on the modeling field. A Bezier curve is constructed from these points, and cells on its path are set into the “fiber” state. After that, the fiber increases its diameter to the specified value.

**Figure 13:**
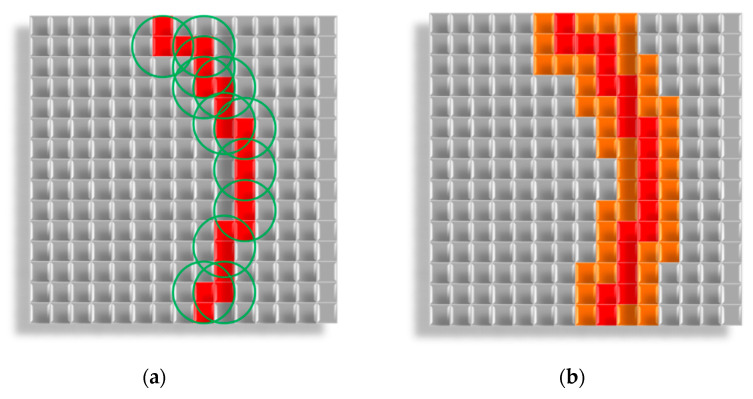
Creation a fiber with Bezier curve: 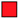
—“fiber” cells,
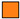
—“fiber” cells, added after diameter increase, 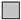
—“empty” cells, 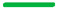
—circles, formed around “fiber” cells: (**a**) Forming circles around “fiber” cells; (**b**) Changing states of cells inside circles into “fiber”.

**Figure 14:**
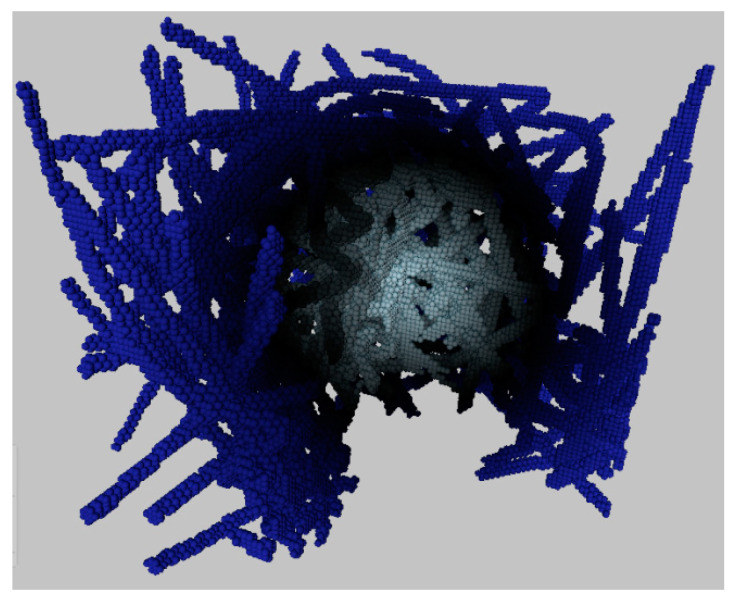
Sectional 3D digital structure of a chitosan-based aerogel.

The algorithm for increasing the fiber diameter works as follows: a sphere (circle for a two-dimensional case) with a diameter equal to the input fiber diameter is formed around each point of the fiber. All cells inside the sphere (circle) are set into the “fiber” state (Figure 13):

Fibers are constructed until the structure reaches specified porosity. Three-dimensional digital copy of aerogel fibrous structure, obtained with developed model is presented in Figure 14.

The proposed model was used to generate digital copies of the structures of experimental samples obtained in Section 2.

The main goal in modeling of nanoporous structures such as aerogels is to obtain a digital copy of the material that corresponds to a specific sample. If the structures of the digital copy and the experimental sample correspond to each other, the digital copy can be used in the future to predict the properties of this sample. They correspond to each other if the deviation of the structure parameters of the digital copy from the structure parameters of the experimental sample does not exceed the specified accuracy. The key structure characteristics of aerogels are specific surface area and pore size distribution (PSD). These parameters influence the structure and aerogel properties such as thermal conductivity, sorption, and mechanical properties. Therefore, in this work, the PSD and the specific surface area were chosen as the criteria for the correspondence between the digital copy and the experimental sample their deviation from the experimental values should not exceed 15%.

The pore size distribution of a digital copy can be represented as a function of the pore volume on the structure porosity and the fiber diameter:

(5) $$V_{\mathrm{pore},D}=f\left(\mathrm{Porosity},\;d\right),$$

where $V_{\mathrm{pore},D}$—volume of pores with diameter D, cm^3^, Porosity—structure porosity, %, d—fiber diameter, nm.

Therefore, the whole pore volume in structure $V_{\mathrm{pore}}$ is equal:

(6) $$V_{\mathrm{pore}}=\sum_{D}V_{\mathrm{pore},D},$$

It can be seen from Equation (5) that the pore size distribution of a digital copy depends on the porosity and fiber diameter, which is directly related to the input parameters of the model.

A similar relationship is true for the specific surface area:

(7) $$S_{\mathrm{ssa}}=g\left(\mathrm{Porosity},\;d\right),$$

where $S_{\mathrm{BET}}$—structure specific surface area, m^2^/g.

The porosity can be directly obtained from experimental data, therefore, during computational experiments, its value was known.

Obtaining experimental data on the diameter of aerogel fibers is a challenging task; therefore, in this work the fiber diameter was a variable parameter that varied in a given range until the deviation of the digital copy pore size distribution from the sample pore size distribution will be below 15%. Thus, considering that the porosity is known, and the fiber diameter d varies, Equations (5) and (7) are reduced to the following form:

(8) $$V_{\mathrm{pores},D}=f\left(d\right)$$

(9) $$S_{\mathrm{BET}}=g\left(d\right)$$

Therefore, in this work, the pore size distribution depended only on one parameter of the fiber diameter d, which was varied.

The method for determining the value of the diameter d is similar to the method of shooting in solving boundary value problems. The diameter d must satisfy the following conditions:

(10) $$\frac{\left|V_{\mathrm{pore},D}^{\exp}-V_{\mathrm{pore},\;D}^{\mathrm{calc}}\left(d\right)\right|}{V_{\mathrm{pore},D}^{\exp}}\;\leq \delta ,\;D\in [1,\;100]$$

(11) $$\frac{\left|S_{\mathrm{BET}}^{\exp}-S_{\mathrm{BET}}^{\mathrm{calc}}\left(d\right)\right|}{S_{\mathrm{BET}}^{\exp}}\;\leq \delta$$

where δ is the maximum deviation, which in this work was taken equal to 15%, and D is the pore diameters that are present in the pore space, $V_{\mathrm{pore},D}^{\exp}$ and $V_{\mathrm{pore},D}^{\mathrm{calc}}$ is the pore volume with a diameter D of the experimental sample and the digital structure, respectively, cm^3^, $S_{\mathrm{BET}}^{\exp}$ and $S_{\mathrm{BET}}^{\mathrm{calc}}$ are the specific surface areas of the experimental sample and the digital structure, respectively, cm^2^/g.

Therefore, the problem statement of modeling nanoporous fibrous structures was as follows:

Two structural parameters were chosen as criteria for comparing a digital copy and an experimental sample: pore size distribution and specific surface area.

The input parameters of the model are the porosity of the structure and the fiber diameter. The porosity is a known value, the fiber diameter is a variable parameter. PSD and specific surface area depend on it.

To obtain a digital copy corresponding to the experimental sample, according to the selected criteria, the fiber diameter d was varied in the range from 18 to 50 nm. The range was selected based on the analysis of SEM images of the aerogel samples.

### 3.2. Digital Structure Parameters Evaluation

Digital structure pore size distribution and specific surface area were evaluated with particular algorithms. To calculate the specific surface area, the total area of free cell faces is calculated (faces that are not adjacent to other “fiber” cells).

**Figure 15:**
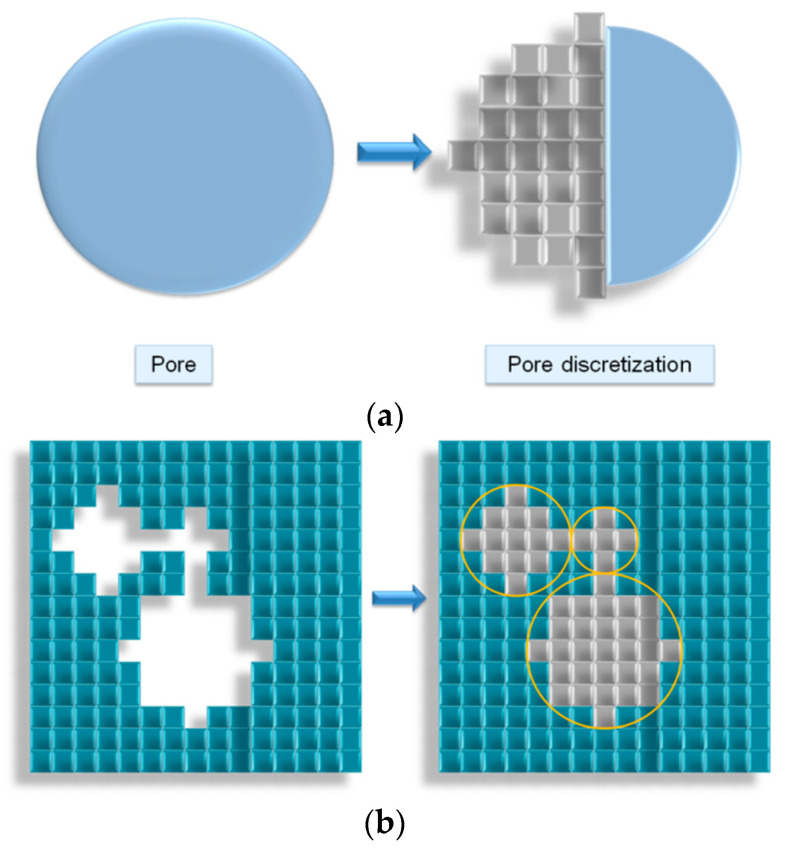
PSD calculation algorithms illustration: (**a**) Cell representation of a pore; (**b**) Placing pores in the form of cells in a digital structure.

Pore size distribution calculation algorithm of the digital structure was previously described in [31]. Its main idea is to fill each cell of the digital structure with balls of certain diameters. If there are no "fiber" cells inside the ball, then the ball is considered to be inscribed, and the volume occupied by the ball is considered to be the volume occupied by the pore of the same diameter. The diameter of the balls changes from maximum to minimum with a given step, so that a situation when a large volume is filled with small balls is impossible. The calculation goes until the diameter of the inscribed balls reaches a diameter equal to the size of one cell. Figure 15 shows an illustration of the operation of the algorithm for a two-dimensional case, but the algorithm can calculate PSD for three-dimensional structures too.

The result of the algorithm is the values of the pores internal volume of different diameters, which are then used to plot the calculated curve of pore size distribution.

However, a direct comparison of the experimental pore size distribution curves with the calculated ones is impossible because the digital structure and the algorithms are discrete, and, therefore, the diameters of the inscribed pores can only be integers and depend on the scale of one cell. Therefore, for a correct comparison, the experimental curves are interpolated to obtain the values of pore volumes for integer values of diameters that correspond to the diameters of the calculated curves. A detailed recalculation explanation is given in [31].

However, it should be noted that, due to the discreteness of the model, the values of diameters of 5 cells and less (10 nm and less for a scale of one cell of 2 nm) were not compared in this work, since in this case the pores cannot be represented with required accuracy.

## 4. Results

To generate digital structures corresponding to the experimental samples, computational experiments were carried out using a model based on the Bezier curves.

It should be noted that the pore volume of the area considered in this work is less than the total pore volume of the sample because of the pores’ presence that are not measured correctly using nitrogen porosimetry. Therefore, the porosity used as an input parameter during generation was calculated as follows [31]:

(12) $$V_{\mathrm{solid}}=\left(1-П\right)V_{\mathrm{sample}}$$

(13) $$V_{\mathrm{sample}}=m/\rho_{\mathrm{bulk}}$$

where $V_{\mathrm{solid}}$—solid part sample volume, cm^3^, П—sample porosity, $V_{\mathrm{sample}}$—sample volume, cm^3^, m—sample mass, g, $\rho_{\mathrm{bulk}}$—sample bulk density, g/cm^3^. Pore volume is calculated as the follows:

(14) $$V_{\mathrm{pore}}=V_{\mathrm{nit}}\cdot m$$

where $V_{\mathrm{nit}}$—pore volume per mass, obtained with BJH from desorption curve, cm^3^/g. Therefore, porosity of sample parts that are used as the model input parameter is calculated using the following correlation:

(15) $${\mathrm{Porosity}}_{\mathrm{nit}}=\frac{V_{\mathrm{pore}}}{V_{\mathrm{solid}}+V_{\mathrm{pore}}}=\frac{V_{\mathrm{nit}}}{\left(1-P\right)/\rho_{\mathrm{bulk}}+V_{\mathrm{nit}}}$$

**Figure 16:**
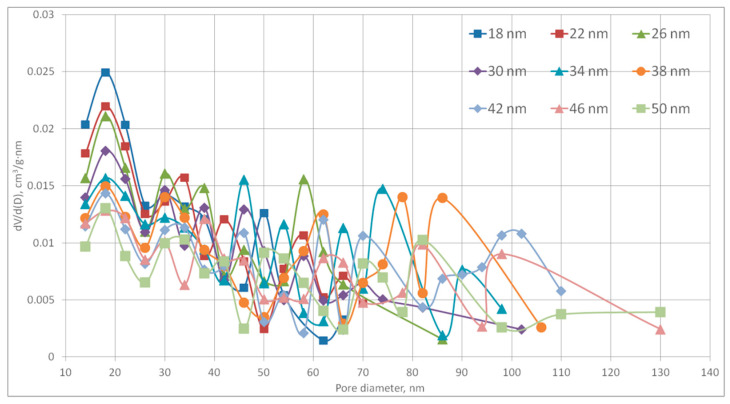
Pore size distribution of digital structures with 65% porosity and different fiber diameter values.

The porosity value ${\mathrm{Porosity}}_{\mathrm{nit}}$ was used as an input parameter to generate the digital structures. Nine structures with different fiber diameters were generated for each experimental sample. Among generated structures was selected the one which that had the smallest deviation of the pore size distribution and specific surface area from the experimental sample. Figure 16 shows the calculated pore size distribution curves for different values of the fiber diameter for a structure with a porosity of 65% in a field of 160 × 160 × 160 cells at a 2 nm scale of one cell. Pores with 10 nm diameter and below were not considered, since the model does not allow measuring their volume with sufficient accuracy.

Figure 16 shows that at constant porosity with increasing fiber diameter, the peaks of the curves shift towards larger diameters. All structures were generated on a 160 × 160 × 160 cells field, the scale of one cell was 2 nm. Thus, the dimensions of the generated sections of the structures are 320 × 320 × 320 nm. In addition, since the model is discrete, the fiber can only increase its diameter in 2 cells (4 nm) steps. Fiber number depends on porosity and fiber diameter and varied with different input parameters of the model.

**Figure 17:**
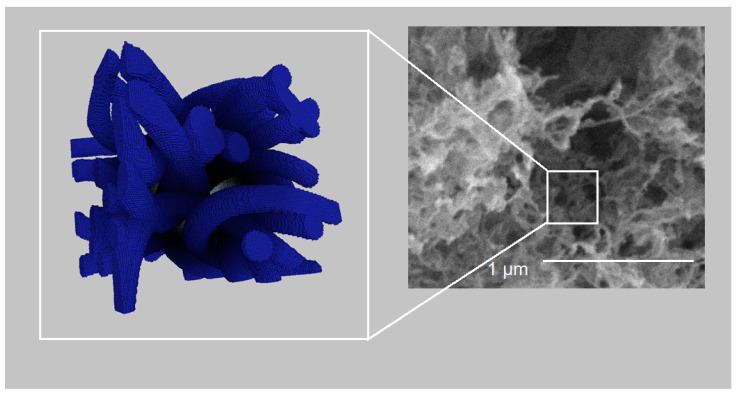
Visual comparison between three-dimensional digital copy and SEM image of sample 2.

**Figure 18:**
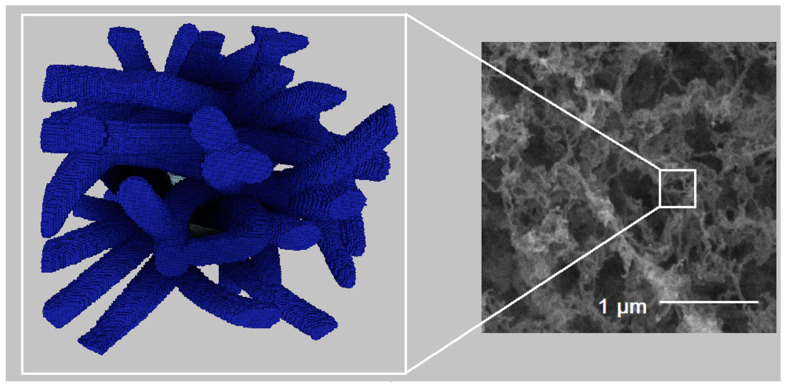
Visual comparison between three-dimensional digital copy and SEM image of sample 6.

Figure 17 and Figure 18 show visual comparison between digital structures and SEM images for samples 2 and 6, respectively.

Digital structures region with size 320 × 320 × 320 nm were generated. Digital structures contain a network of cross fibers. This scale allows for considering the contribution of each fiber in structure properties.

The correspondence between the digital structure and the experimental sample is determined in accordance with the selected criteria of comparing: the pore size distribution and the specific surface area.

**Figure 19:**
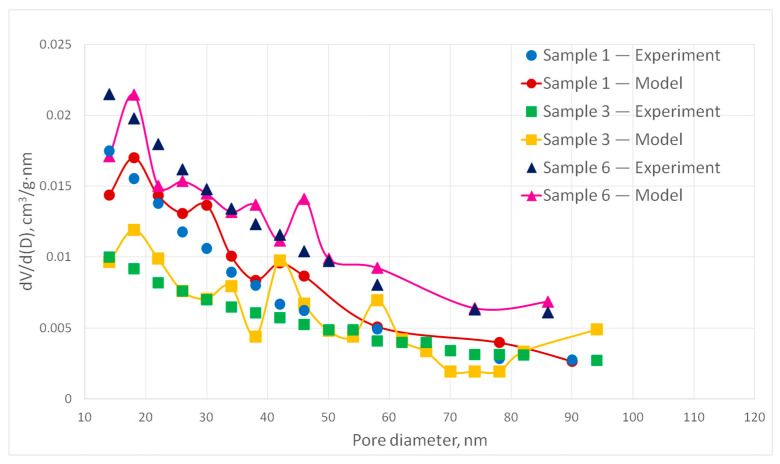
Experimental and corresponding digital structures PSD curves for samples 1, 3 and 6.

**Figure 20:**
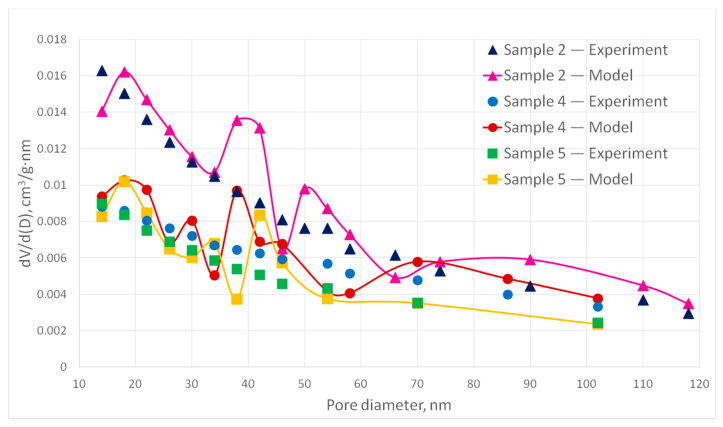
Experimental and corresponding digital structures PSD curves for samples 2, 4 and 5.

Figure 19 and Figure 20 show experimental samples and the corresponding digital structures comparison of the pore size distribution for samples 1–6. 

The deviation of the calculated values of the pore size distribution curves from the experimental ones does not exceed 15%. However, it should be noted that some points on the curve have a deviation of more than 15%, which is due to the stochastic nature of the model. Therefore, it can be concluded that the generated digital structures correspond to the experimental samples. It can be seen that pore size distribution significantly depends on porosity and fiber diameter as was intended. In Figure 19, sample 6 has the largest porosity and small fiber diameter. Its PSD curve paths above sample 1 and 3 curves. Sample 1 has the same fiber diameter 30 nm but lower porosity. Its PSD curve is similar to sample 6 but goes lower. At the same time sample 3 has the biggest fiber diameter and the smallest porosity. Its curve is different from curves of sample 1 and 3. In the Figure 20, sample 2 has small fiber diameter and big porosity, so its curve paths higher, and pores with large diameters are indicated. Samples 4 and 5 fiber diameters are close to each other, but sample 4 has higher porosity, so it can be noted that sample 4 has more pores with high diameters.

Table 3 shows a comparison of the specific surface area of the experimental samples and the corresponding digital structures.

The deviation of the calculated values of the specific surface area from the experimental ones does not exceed 11%. Based on the data obtained, it can be concluded that the generated digital structures correspond to the experimental samples.

## 5. Discussion

In this work, 16 samples of aerogel particles based on chitosan were obtained to the method described in Section 2. For these samples, their structure characteristics were obtained using nitrogen porosimetry: pore size distribution curves were obtained using the BJH method, and the specific surface area values were obtained using the BET method. Skeletal and bulk densities were obtained using helium pycnometry.

An original computer model was developed to generate nanoporous fibrous structures based on Bezier curves.

The proposed model was used in computational experiments to generate the corresponding digital structures. For each of the 16 experimental samples, 9 digital structures with different fiber diameters were generated. Thus, 144 computational experiments were carried out, as a result of which the dependence of the pore size distribution of digital structures on the parameters of the model was investigated and digital copies of chitosan-based aerogels were obtained.

Then, generated digital structures and experimental samples were compared. The pore size distribution curves and the specific surface area were selected as criteria of comparing. The digital copy corresponded to the experimental sample if the deviation of the calculated values of these parameters from the experimental ones did not exceed 15%. For each experimental sample, a corresponding digital structure was found. The deviation of the calculated pore size distribution curves for all samples does not exceed 15%, and the deviation of the specific surface area does not exceed 11%. Therefore, it can be concluded that digital copies correspond to the experimental samples of chitosan aerogel, and the proposed model can be used in the future to generate digital copies of nanoporous fibrous structures.

## 6. Conclusions

In this work, the new model for generating digital copies of fibrous porous structures were developed. The developed model is based on Bezier Curves and allows generating fibers with shape, corresponding to the real ones.

The digital copies of chitosan-based aerogels structures were generated. The obtained digital copies and the experimental structures were compared by two parameters: pore size distribution and specific surface area. Digital structures, pore size distribution, and specific surface area corresponded to the experimental ones, so digital copies correspond to experimental samples of chitosan aerogel.

The developed model allows generation of digital copies corresponding to experimental nanoporous fibrous structures samples, such as chitosan-based aerogels. The generated digital copies can be used with models which predict different properties of the aerogel by its structure such as thermal conductivity and mechanical properties. It will reduce the number of experimental studies in developing aerogels with required properties.

The obtained digital structures of chitosan-based aerogels will be used to generate digital structures with embedded active pharmaceutical ingredients (API). These digital structures will be used to predict the release of APIs, which will reduce the number of required experiments when creating new drugs using aerogels, based on chitosan, as a carrier. This model can be implemented using high-performance parallel computing, which will significantly increase the efficiency of calculations.

## Figures and Tables

**Table 1:** Variable parameters in the preparation of chitosan aerogel particles.

Sample №	C_chitosan_, Mass%	C_acid_, M	C_alkali_, M	C_polymer_, Mass%
1	1	0.1	1	0
2	1	0.2	1	0
3	2	0.1	1	0
4	2	0.2	1	0
5	1	0.1	1	0.05
6	1	0.2	1	0.05
7	2	0.1	1	0.05
8	2	0.2	1	0.05
9	1	0.1	0.1	0
10	1	0.2	0.1	0
11	2	0.1	0.1	0
12	2	0.2	0.1	0
13	1	0.1	0.1	0.05
14	1	0.2	0.1	0.05
15	2	0.1	0.1	0.05
16	2	0.2	0.1	0.05

**Table 2:** Analitical studies result of the chitosan-based aerogel particles samples.

Sample №	S_BET_, m^2^/g	V_pore_, cm^3^/g	ρ_skeletal_, kg/m^3^	ρ_bulk_, kg/m^3^	Porosity, %
1	301 ± 2.13	1.28	2050	55	98.27
2	270 ± 2.11	1.32	1909	48	97.44
3	166 ± 1.99	0.74	1893	66	96.49
4	192 ± 2.12	1.07	1842	86	95.30
5	151 ± 2.15	0.07	1880	34	98.16
6	360 ± 2.15	1.80	2367	38	98.35
7	143 ± 1.87	0.79	2129	67	96.84
8	237 ± 2.17	1.43	1741	62	96.40
9	261 ± 2.16	1.50	1743	52	97.01
10	323 ± 1.96	2.25	1788	53	96.99
11	347 ± 2.01	2.26	1676	64	98.13
12	135 ± 2.06	0.93	2232	52	97.63
13	248 ± 1.98	1.72	1933	49	97.44
14	227 ± 2.03	1.39	3307	39	98.80
15	190 ± 2.11	1.28	1778	54	96.93
16	175 ± 2.07	1.10	2247	49	97.81

**Table 3:** Experimental and digital structures SSA comparison.

Sample №	Fiber Diameter, nm	Porosity_nit_, %	S_BET exp._, m^2^/g	S_BET dig._, m^2^/g	Deviation, %
1	30	67	301	293	3
2	34	68	270	280	4
3	42	55	166	161	3
4	46	64	192	176	8
5	42	54	151	168	11
6	30	74	360	343	5
7	34	53	143	137	4
8	30	77	237	229	3
9	30	65	261	258	1
10	34	64	323	317	2
11	34	72	347	340	2
12	42	66	135	128	5
13	26	67	248	256	3
14	34	73	227	213	6
15	34	58	190	187	1
16	38	67	175	191	9
